# Molecular epidemiologic characteristics of hemagglutinin from five waves of avian influenza A (H7N9) virus infection, from 2013 to 2017, in Zhejiang Province, China

**DOI:** 10.1007/s00705-021-05233-5

**Published:** 2021-09-30

**Authors:** Yi Sun, Haiyan Mao, Xiuyu Lou, Xinying Wang, Yin Chen, Biaofeng Zhou, Yanjun Zhang

**Affiliations:** 1 Zhejiang Provincial Center for Disease Control and Prevention, 3399 Binsheng Road, Hangzhou, 310051 Zhejiang China; 2 Zhejiang Workstation for Emerging Infectious Disease Control and Prevention, Chinese Academy of Medical Sciences, Hangzhou, Zhejiang China

## Abstract

There have been five waves of influenza A (H7N9) epidemics in Zhejiang Province between 2013 and 2017. Although the epidemiological characteristics of the five waves have been reported, the molecular genetics aspects, including the phylogeny, evolution, and mutation of hemagglutinin (HA), have not been systematically investigated. A total of 154 H7N9 samples from Zhejiang Province were collected between 2013 and 2017 and sequenced using an Ion Torrent Personal Genome Machine. The starting dates of the waves were 16 March 2013, 1 July 2013, 1 July 2014, 1 July 2015, and 1 July 2016. Single-nucleotide polymorphisms (SNPs) and amino acid mutations were counted after the HA sequences were aligned. The evolution of H7N9 matched the temporal order of the five waves, among which wave 3 played an important role. The 55 SNPs and 14 amino acid mutations with high frequency identified among the five waves revealed the dynamic occurrence of mutation in the process of viral dissemination. Wave 3 contributed greatly to the subsequent epidemic of waves 4 and 5 of H7N9. Compared with wave 1, wave 5 was characterized by more mutations, including A143V and R148K, two mutations that have been reported to weaken the immune response. In addition, some amino acid mutations were observed in wave 5 that led to more lineages. It is necessary to strengthen the surveillance of subsequent H7N9 influenza outbreaks.

**Supplementary Information:**

The online version contains supplementary material available at 10.1007/s00705-021-05233-5.

## Introduction

The first case of avian influenza A/H7N9 virus infection in humans was reported in 2013 in eastern China [4]. Since then, five epidemic waves, as well as sporadic cases, have emerged. To date, 1568 confirmed human H7N9 infections have been reported, of which approximately 39% were fatal, with most cases occurring in mainland China (March 2021, Food and Agriculture Organization of the United Nations http://www.fao.org/ag/againfo/programmes/en/empres/h7n9/situation_update.html). Global concerns persist because highly pathogenic H7N9 variants emerged in wave 5 [7]. The H7N9 virus continues to be detected in animals and the environment. Therefore, additional sporadic human cases of avian influenza A (H7N9) that have not yet been reported are expected in Chinese provinces.

The viral hemagglutinin (HA) facilitates virus entry into host cells and is also the principal target of the adaptive immune response against influenza virus [1]. The structure of the receptor binding site (RBS) of H7 HA is similar to that of all published influenza A virus HAs and is composed of several structural elements, including the 190-helix, 130-loop, 140-loop, 150-loop, and 220-loop [18]. Several studies investigating the evolutionary relationships of the HA segment of H7N9 have been reported, with a wide range of sample sizes from different areas and mostly using samples from multiple waves of H7N9 since 2013 [8, 9, 11, 12, 14, 15] . The Yangtze River Delta and The Pearl River Delta are two regions that experienced outbreaks and epidemics of H7N9 during epidemic waves 1–5 [15]. The first wave emerged in the Yangtze River Delta, and subsequently, the spatial range of H7N9 human cases increased, along with lineage alterations during epidemic wave 2 [17]. One lineage of wave 2 spread to the Pearl River Delta, which led to epidemics of several lineages in waves 3–5 [15]. Lineages of wave 5 from the Yangtze River Delta and Pearl River Delta have been different [6]. For example, a highly pathogenic H7N9 strain in which the amino acid residues RKRT were inserted at the cleavage site of the peptide region connecting HA1 and HA2 was found in the Pearl River Delta, but not in the Yangtze River Delta [7].

Zhejiang Province is considered one of the likely areas for the emergence of H7N9 in the Yangtze River Delta, although the virus was first recognized in Shanghai (A/Shanghai/1) [4, 17]. Previously, we reported the epidemiological characteristics of the five epidemic waves of H7N9 infection in Zhejiang Province, concluding that the epidemic cross-regional expansion continued and mostly occurred in rural areas [16]. However, comprehensive molecular genetics research based on these samples from Zhejiang was lacking. In this study, we sequenced the genomes of 154 H7N9 virus samples from the five waves and analyzed the molecular genetic characteristics of each H7N9 strain from different waves. Moreover, we investigated the phylogenetic pattern and dynamic evolution of H7N9 in Zhejiang since it emerged in 2013, which may contribute to the prevention and control of H7N9 in the future.

## Materials and methods

### Sample collection and viral RNA extraction

This study was approved by the ethics committee of the Zhejiang Provincial Center for Disease Control and Prevention (ZJCDC), China. Following the ‘Surveillance Program of Influenza in China’ published by the National Health Department, oropharyngeal or sputum samples were collected from suspected human cases. Environmental samples and oropharyngeal and cloacal swabs from apparently healthy or diseased poultry in live-poultry markets and poultry farms located close to the patients were also collected. Viral RNA was extracted using a Viral RNA Purification Kit (QIAGEN). Clinical samples were tested by quantitative reverse-transcription polymerase chain reaction (qRT-PCR) using avian influenza H7N9 detection kits (Mabsky Bio-tech Co., Ltd. and Zhengzhou Zhongdao Biotechnology Co., Ltd.), following the manufacturers’ instructions.

### Genome sequencing

After preparation of viral RNA, libraries were constructed consisting of random fragments averaging 200 base pairs (bp) in length. Each sample was assigned a different barcode using an Ion Xpress Plus Fragment Library Kit (Life Technologies). We then pooled the libraries and sequenced them using an Ion Torrent Personal Genome Machine (v 4.2.1). Next, we used Torrent Suite software to perform quality control under default parameters. Specifically, a sliding-window-based method was used to trim reads from the 3' end according to the average base quality value.

### Phylogenetic analysis of the HA gene

HA gene sequences were edited using Geneious (Version 11.1.5). The reference strains were identified via BLAST comparisons of the H7N9 HA gene sequences to sequences in the GISAID database (https://www.gisaid.org/). Full-length HA sequences of strain Anhui/1/2013, used as the reference strain, were downloaded from GenBank and GISAID. A multiple sequence alignment was performed using MUSCLE [3]. A phylogenetic tree was constructed using RAxML [13] with GTRGAMMA as the nucleotide substitution model, and 1,000 bootstrap replicates were run to assess the reliability of the phylogenetic trees. In general, distinct lineages with bootstrap values larger than 80% were visible in the phylogenetic tree.

### Single-nucleotide polymorphism (SNP) analysis

SNP analysis was performed using the online analysis module (http://www.fludb.org) for analyzing sequence variation in the Influenza Research Database [19, 20], with 154 HA nucleotide sequences in fasta format as input. We calculated the score at each nucleotide position according to the formula [2]

$$ S = - C\sum\nolimits_{i = 1}^{n} {pi \log 2\left( {pi} \right)} $$

where *Pi* is the frequency of the *i*-th allele, and the constant *C* value is 100. For nucleotides, scores can range from 0 (no polymorphism) to 232 (four alleles and an indel; 20% frequency each). Sites with scores greater than or equal to 10 were extracted and visualized together with the wave information of the samples using R script.

### Entropy calculation

The nucleic acid sequences on which multiple sequence alignment had been performed were first translated into amino acid sequences and confirmed using the annotation program of GenBank (https://www.ncbi.nlm.nih.gov/genomes/FLU/annotation/). The entropy value of each amino acid position was calculated using the same formula that was used for the SNP analysis [2, 20], treating 154 sequences as a population. For amino acids, scores (entropy) can range from 0 (no polymorphism) to 439 (20 alleles and an indel, 4.7% frequency each). Finally, we defined the amino acid sites with entropy greater than 60 as highly variable sites and extracted and visualized them for SNP analysis.

## Results

### Molecular epidemiologic characteristics in the five waves

From March 2013 to June 2017, there were a total of 320 H7N9 human infections, including 116 fatal cases, reported in Zhejiang Province, China. The five waves began on 16 March 2013, 1 July 2013, 1 July 2014, 1 July 2015, and 1 July 2016. A total of 199 samples (30, 33, 39, 18, and 79 samples for waves 1–5) were sequenced. We finally obtained 154 full HA sequences of H7N9 (23, 29, 29, 17, and 56 samples for waves 1–5) from different epidemic waves since 2013. In each wave, the number of samples from humans, the environment, and chickens were 21/2/0, 22/4/3, 18/11/0, 13/4/0, and 47/9/0, respectively (Table 1). The nucleotide sequence identity of the H7N9 strains from waves 1–5 in Zhejiang Province in 2013–2017 to strain A/Anhui/1/2013(H7N9) was 99.8%, 99.5%, 98.8%, 98.1%, and 98.7%, respectively; the amino acid sequence identity was 100%, 99.9%, 99.4%, 99.1%, and 99.6%, respectively.

**Table 1:** An overview of 154 samples from five waves

Wave	Human	Environment	Chicken	Total
1	21	2	0	23
2	22	4	3	29
3	18	11	0	29
4	13	4	0	17
5	47	9	0	56

**Fig. 1:**
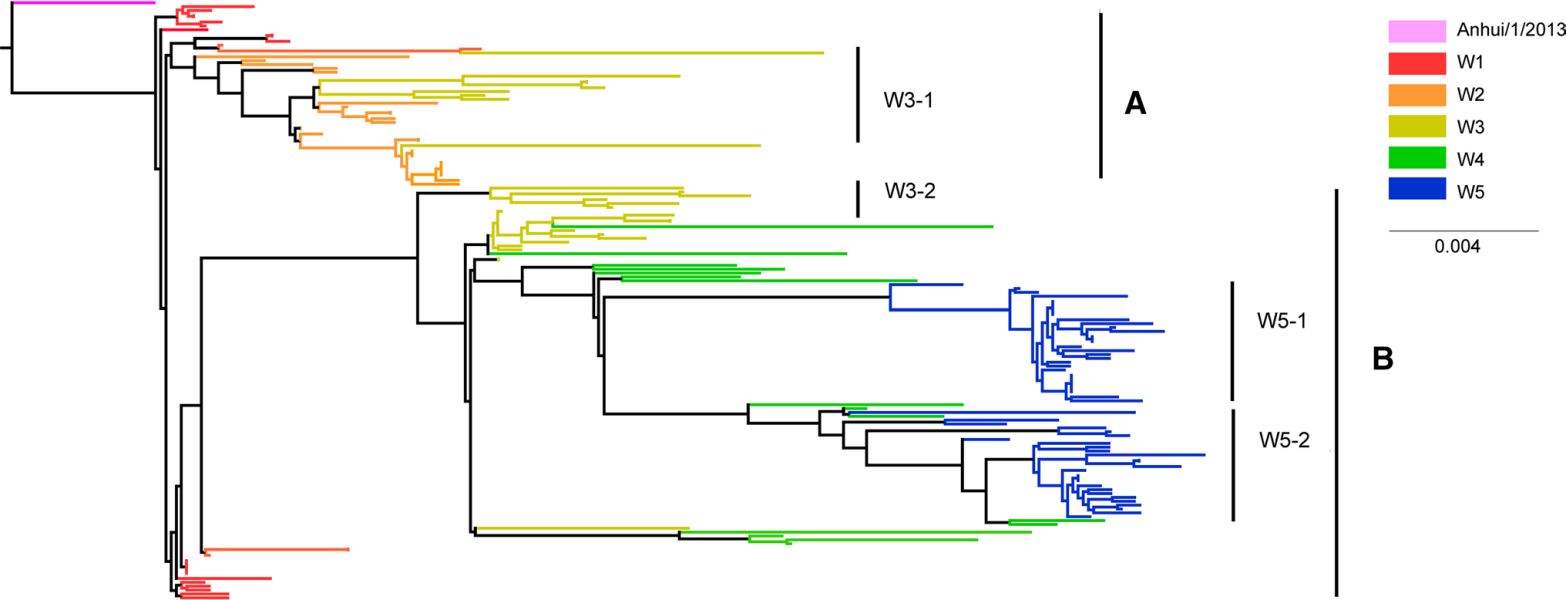
Phylogenetic analysis of the HA gene of the H7N9 virus during epidemic waves 1–5 in Zhejiang Province. Maximum-likelihood trees were constructed with RAxML, applying the GTRGAMMA nucleotide substitution model with 1000 bootstrap replicates and A/Anhui/1/2013 as the root. Samples from different waves are indicated by different colors.

**Fig. 2:**
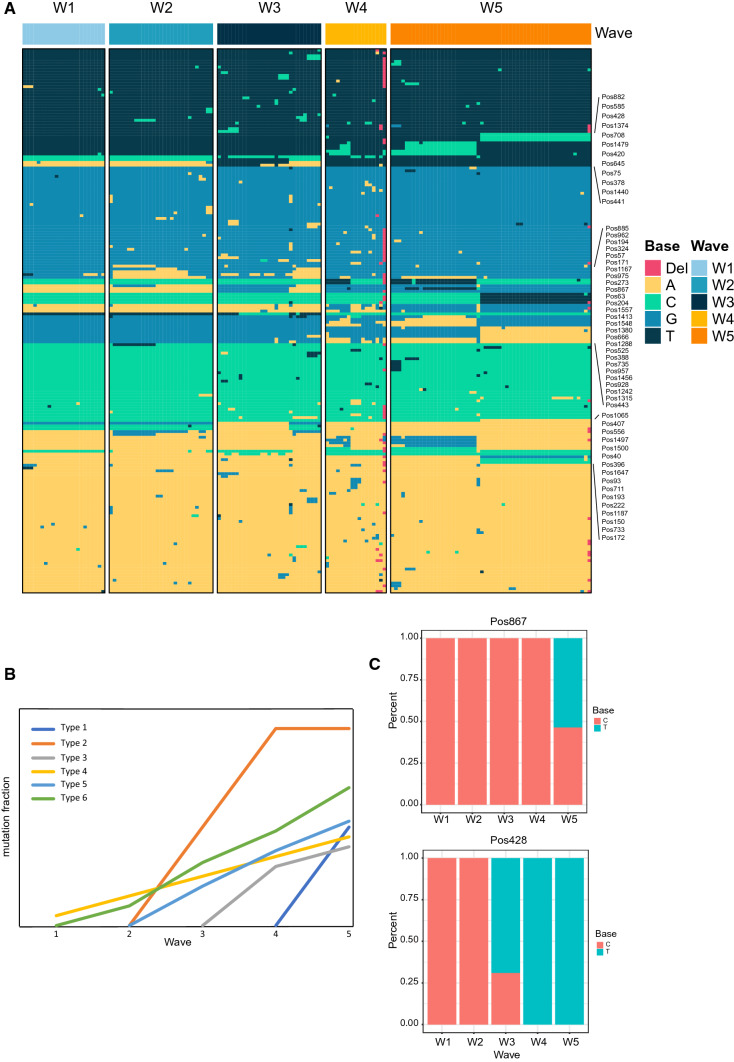
Single-nucleotide polymorphisms (SNPs) of HA genes in the five epidemic waves of H7N9 virus in Zhejiang Province. **A** Overview of all nucleotide sites whose SNP score is larger than 10. A, T, C, G, and deletions are indicated by different colors. **B** The six types of SNPs identified among the five epidemic waves. **C** The SNPs of two nucleotide sites (position 867 and position 428) of the HA gene in five epidemic waves.

**Fig. 3:**
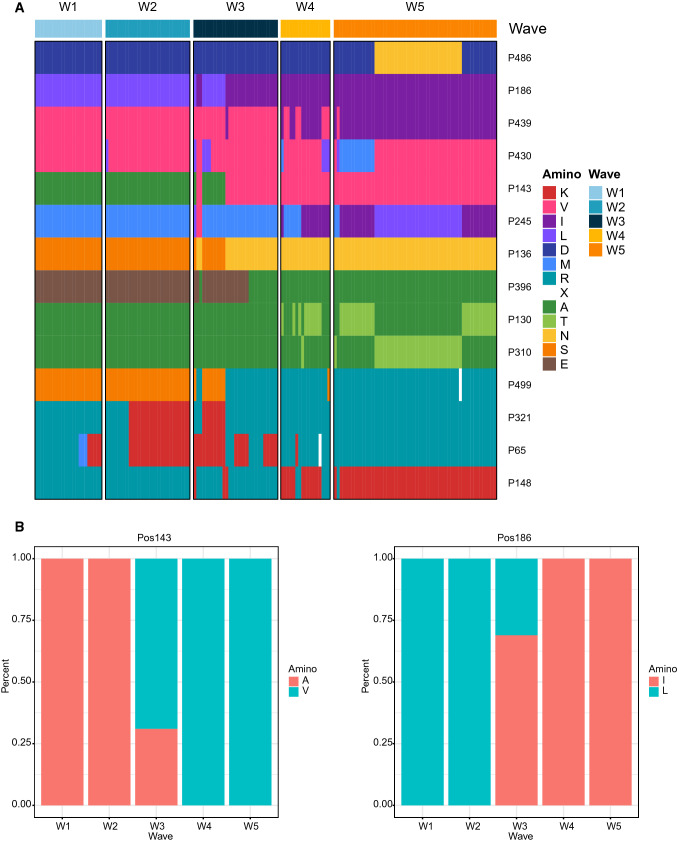
Amino acid sequence variation in the HA protein in the five epidemic waves of H7N9 virus in Zhejiang Province. **A** Amino acid residues with entropy greater than 60 were chosen as highly variable sites. Different amino acids are indicated by different colors. **B** Distribution of two amino acid residues (position 143 and position 186) of the HA gene in five epidemic waves.

We performed phylogenetic analysis on the HA genes of these virus samples. The phylogenetic tree showed an obvious evolutionary relationship from wave 1 to wave 5 (Fig. 1). In addition, two clear evolutionary branches could be clearly seen. Branch A included half of the sequences from waves 1 and 3 and almost all of the sequences from wave 2. The remaining sequences from waves 1 to 3 and from waves 4 and 5 constituted branch B. What stands out is that wave 3 was separated into two clades, which were located in two main branches, suggesting that wave 3 played a significant role in transmission and in the transition from waves 1 and 2 to waves 4 and 5. A similar phenomenon can also be observed in the subsequent analysis of SNPs and amino acid variation (Figs. 2 and 3). In addition, in branch B, samples from wave 5 were further clustered into two clades with a comparable scale but with major differences, indicating that two stable strain subtypes may have been formed in wave 5.

### The single-nucleotide polymorphisms (SNPs) of HA genes in the five waves

We used the SNP module of the Influenza Research Database to calculate the SNP score at each nucleotide position, using a total of 154 sequences. SNPs with scores greater than 10 were chosen, and their distribution in the five waves is displayed in Fig. 2A and Supplementary Table S1. We found that 72% (139/194) of the SNPs failed to show a significant correlation with waves (Chi-square test *p* > 0.05), while 28% (55/194) of SNPs exhibited a regular dynamic with the waves and were classified into six types (Fig. 2B). The mutation fractions of SNPs among the waves all showed an increasing trend with time, but with different origins, ranging from wave 1 to wave 5. SNPs of type 1 were referred to as wave-5-specific mutations (Fig. 2C). These SNPs are representative of the lineage divergence among samples from wave 5. SNPs of type 2 represent the key mutations leading to lineage divergence in wave 3 (Fig. 2C). The other four types demonstrate the evolutionary trajectory among the waves. These SNPs made an important contribution to the branching of the phylogenetic tree, resulting in consistent clustering of the sequences. When compared with the phylogenetic tree, the analysis of SNPs allowed specific mutations associated with lineage divergence to be identified.

### The amino acid sequence variants of HA genes in the five waves

The amino acid sequence of HA is closely related to the functions of the protein, such as viral transmissibility, receptor binding specificity, pathogenicity, and virulence [5], so we focused on the mutation of amino acid residues. Similar to SNPs, the polymorphism of each amino acid residue can be analyzed by entropy, a concept used to quantify the mutation of each amino acid residue. The only difference between the formulas for the SNP score and entropy is the constant. Taking 154 amino acid sequences as a population, we determined the entropy value of each amino acid residue. A total of 14 sites had entropy values greater than 60 and were therefore identified as highly variable sites (Fig. 3A). These 14 sites displayed a strong correlation with the waves (Fisher's exact test *p* < 0.05). As shown in Fig. 3 and Table 2, there were three major strains in wave 5, judging from the 14 amino acid mutations detected. Wave 1 and wave 2 were identical in 12 of the sites, differing only at residues 321 and 65. The differences between waves 1 and 2 and wave 5 were mainly concentrated in the amino acid residues 186, 439, 143, 136, 245, 396, 499, and 148. Wave 3 and wave 4 showed variation at several sites, and wave 3 might have played a transitional role from waves 1 and 2 to wave 5. We also observed some wave-specific mutations. For example, the amino acid mutations D486N, V430M, M245L, A130T, and A310T mainly occurred in wave 5. Some of these mutations alter the polarity of the amino acid at that position. For example, a hydrophilic amino acid was replaced by a hydrophobic one in the case of the E396A mutation, while hydrophobic amino acids were replaced by hydrophilic ones in the A130T and A310T mutations.

**Table 2:**
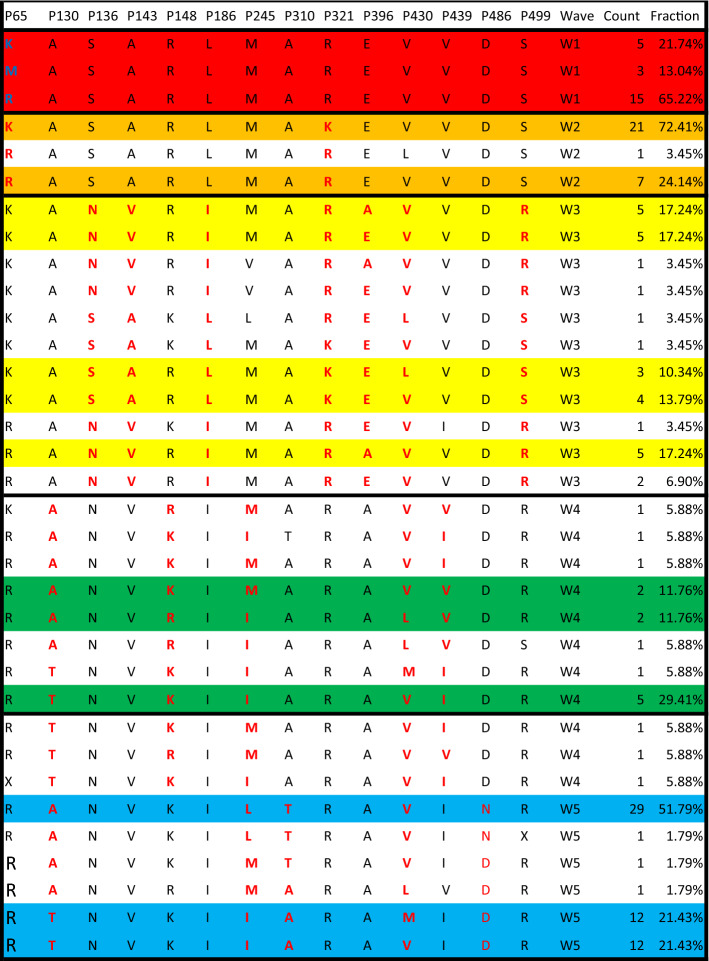
The dominant amino acid variants of the HA genes in the five epidemic waves of H7N9 virus in Zhejiang Province

After identifying 14 amino acid residues with high mutation frequency in the five waves, we further identified the dominant subtypes in each wave (Table 2). It is apparent that there were significant differences in the dominant subtypes among waves. Wave 3 had five main subtypes (>10%), and five subtypes represented less than 10% of the total. Wave 4 was similar to wave 3 but had only three main subtypes (>10%). Waves 1, 2, and 5 had 2–3 main subtypes, and the three main subtypes can be seen especially clearly in wave 5. These results are consistent with the results of phylogenetic analysis.

The number of highly variable amino acid residues that we identified was significantly lower than the number of SNPs, mainly because of codon degeneracy. However, amino acid variations are presumably more biologically significant. In addition to these 14 highly variable sites, we also focused on the key mutation sites reported by previous researchers (Supplementary Table S2). The results showed variation at positions 169 and 235, but their entropy values were lower than 60, suggesting that their mutation frequency was not high in this data set. For example, our data set for amino acid residue 235 includes seven I and 147 L residues, which is different from samples from Guangdong (235Q) analyzed in a previous study [7]. The difference in this site may be due to the fact that the samples in this study were limited to Zhejiang Province, where the reassortments and mutations of H7N9 viruses are distinct from those in Guangdong Province.

Given that the RBS of HA is associated with virus adsorption to host cells, we inspected the amino acid sequence variation in the five domains of the RBS. Variable sites were found in each domain, and the 220-loop had the most variable sites, followed by the 150-loop (Table 3). However, only the amino acid substitutions A143V in the 130-loop and R148K in the 140-loop were among the 14 amino acid residues with high mutation frequency. The amino acid substitution A143V emerged in wave 3 and was sustained through waves 4 and 5 (Fig. 3B). The distribution of the amino acid substitution R148K is similar to that of A143V, but some non-mutated samples emerged in wave 4 and wave 5 with a decreasing trend.

**Table 3:** The entropy for each amino acid residue in the RBS region of HA in the five waves of the H7N9 virus

H7 numbering	H3 numbering	Region	Entropy
139	131	130 loop	0
140	132		0
141	133		0
142	134		0
143	135		96.863
144	136		0
145	137		5.652
146	138		0
148	140	140 loop	99.403
149	141		0
-	142		/
150	143		0
151	144		0
152	145		0
163	156	150 loop	0
164	157		11.299
165	158		0
168	159		5.652
169	160		16.939
199	190	190 helix	0
200	191		0
201	192		0
202	193		13.851
203	194		0
204	195		0
205	196		0
228	219	220 loop	0
229	220		13.851
230	221		0
231	222		21.274
232	223		5.652
233	224		0
234	225		5.652
235	226		26.677
236	227		5.652
237	228		0

## Discussion

Since the emergence of the H7N9 influenza virus, there have been five epidemic waves in Zhejiang Province from 2103 to 2017. A phylogenetic tree based on the HA gene sequences of the H7N9 viruses from the five waves revealed that the viruses causing waves 2 and 3 were derived from two genotypes from wave 1 and evolved into two different lineages. Wave 3 was particularly crucial, as it was the end of branch A as well as the start of branch B. This conclusion was supported by the identification of various SNPs and amino acid mutations in wave 3. A possible explanation is that the virus of wave 3 mutated in the process of spreading to the southern and central areas from northern Zhejiang after the second wave [16]. Wave 5 was considered the most serious of the five epidemic waves of H7N9, as it was epidemic across multiple regions in China with both a high incidence and a high fatality rate [14]. It is known that highly pathogenic avian influenza viruses first emerged in 2016 in Guangdong [19]. However, we failed to find those strains in this study, suggesting that the wave 5 H7N9 virus in Zhejiang is of a different subtype. This conclusion is consistent with that of a previous study showing that there were four subtypes in wave 5, with three in the Pearl River Delta region and one in the Yangtze River Delta region [14]. Notably, the virus had evolved further into two different primary lineages in wave 5, implying that mutation of H7N9 is still occurring and that ongoing surveillance of H7N9 is required.

In this study, we identified 55 SNPs in a regular dynamic pattern with the epidemic waves. These SNPs were classified into six types according to their frequency as the waves changed. Two types of SNP that were detected only in wave 3 (428, 585, 273, 666, 407, 556, 1187) or wave 5 (441, 378, 867, 63, 204, 1557, 957, 1456, 1065, 150, 172) made a great contribution to the divergence of lineages in those two waves. The SNPs that were unique to wave 3 brought about a lineage alteration from waves 1 and 2 to waves 4 and 5, while the unique SNPs in wave 5 may reflect the future direction of mutation in H7N9. The other four types of SNP were distributed in more than two waves in different proportions. For example, the fraction of A1413G increased from wave 1 to wave 5, demonstrating the dynamic changes among waves. To summarize, the SNPs we identified not only characterized the five waves of H7N9 but also revealed their dynamic features.

The 14 amino acid sites with high mutation frequency were quite interesting. Eight of the sites were identified in a previous study [10], whereas those at positions 486, 245, 430, 310, 321, and 65 were not among the previously known mutations. A143V and R148K attracted our attention because they are located in a key region of the RBS of the HA gene. Ning et al. analyzed the median infectious dose (ID_50_) of several variants, including 11 of the 14 variants detected in this study, and found that strains containing the mutations A143V and R148K displayed a fourfold lower median susceptibility to neutralization by an antiserum against influenza virus strain A/Anhui/1/2013 in 2018 [10], suggesting that these two mutations significantly influenced immunogenicity. In this study, these two key mutations first emerged in wave 3, initially present in a small fraction of isolates, but soon becoming dominant in waves 4 and 5 with a frequency of 100% and 98%, respectively. This suggests that the fifth or even subsequent epidemic waves of the H7N9 virus may challenge the effectiveness of the vaccine designed for A/Anhui/2013 if H7N9 continues to cause outbreaks in the future.

It is worth mentioning that A310T, D486N, and V430M, which were not identified by Ning et al., were all found in wave 5. A310T and D486N distinguished two subgroups of wave 5, indicating that they may also contribute to the lineage divergence of wave 5. V430M was the only mutation with high frequency in the W5-2 subgroup, implying that viruses based on this lineage may emerge in the future if H7N9 continues to cause outbreaks after wave 5. Therefore, it may be necessary to study the influence of A310T, D486N, and V430M on the immunogenicity of the virus in future.

In summary, in this study, we investigated the molecular characteristics of five waves of H7N9 viruses that occurred in Zhejiang during 2013–2017 and found that the mutation and evolution of H7N9 were in line with the temporality of the waves. Wave 3 played an important role in the outbreak of waves 4 and 5. We identified some characteristic SNPs that changed dynamically with the waves. In addition, among the 14 amino acid mutations with high frequency detected in the five waves, two amino acid mutations, A143V and R148K in the 130-loop and 140-loop, respectively, of the HA RBS region, were the dominant subtypes of wave 5. Previous studies have shown that these mutations may significantly reduce the sensitivity of the virus to neutralizing serum [10]. These 14 amino acid mutations, reflecting the evolutionary direction of the five waves of H7N9, may indeed affect the future evolutionary direction of H7N9 if the virus continues to cause outbreaks. Therefore, these mutations deserve special attention.

## Supplementary Information

Below is the link to the electronic supplementary material.

Supplementary file1 (XLSX 20 kb)

Supplementary file2 (XLSX 23 kb)

Supplementary file3 (XLSX 14 kb)

## Data Availability

All data were deposed in GISAID. Detailed information is available in Supplementary Table 3.
